# *Saccharomyces cerevisiae* Expressing Gp43 Protects Mice against *Paracoccidioides brasiliensis* Infection

**DOI:** 10.1371/journal.pone.0120201

**Published:** 2015-03-19

**Authors:** Mariana Aprigio Assis-Marques, Aline Ferreira Oliveira, Luciana Pereira Ruas, Thaila Fernanda dos Reis, Maria Cristina Roque-Barreira, Paulo Sergio Rodrigues Coelho

**Affiliations:** Departamento de Biologia Celular e Molecular e Bioagentes Patogênicos, Faculdade de Medicina de Ribeirão Preto, Universidade de São Paulo, Av. Bandeirantes 3900, Ribeirão Preto, SP, 14049–900, Brasil; University of Minnesota, UNITED STATES

## Abstract

The dimorphic fungus *Paracoccidioides brasiliensis* is the etiological agent of paracoccidioidomycosis (PCM). It is believed that approximately 10 million people are infected with the fungus and approximately 2% will eventually develop the disease. Unlike viral and bacterial diseases, fungal diseases are the ones against which there is no commercially available vaccine. *Saccharomyces cerevisiae* may be a suitable vehicle for immunization against fungal infections, as they require the stimulation of different arms of the immune response. Here we evaluated the efficacy of immunizing mice against PCM by using *S*. *cerevisiae* yeast expressing gp43. When challenged by inoculation of *P*. *brasiliensis* yeasts, immunized animals showed a protective profile in three different assays. Their lung parenchyma was significantly preserved, exhibiting fewer granulomas with fewer fungal cells than found in non-immunized mice. Fungal burden was reduced in the lung and spleen of immunized mice, and both organs contained higher levels of IL-12 and IFN-γ compared to those of non-vaccinated mice, a finding that suggests the occurrence of Th1 immunity. Taken together, our results indicate that the recombinant yeast vaccine represents a new strategy to confer protection against PCM.

## Introduction

The dimorphic fungus *Paracoccidioides brasiliensis* is the etiological agent of paracoccidioidomycosis (PCM), which is an endemic granulomatous chronic mycosis occurring in Latin America. Epidemiological data indicate that PCM is found with high incidence in Brazil, Argentina, Colombia, and Venezuela [1–4]. Infection occurs by inhalation of fungal spores or particles, which transform into the pathogenic yeast form after reaching the pulmonary alveolar epithelium [5]. Yeast can either be eliminated by immune-competent cells or disseminate to other tissues through lymphatic and hematogenous routes, resulting in a spectrum of clinical manifestations, which vary from asymptomatic, benign and localized to severe and disseminated forms (reviewed in [6]). Clinical and experimental evidence indicate that, similar to other systemic mycosis, Th1 immunity exerts a singular role in the asymptomatic form of PCM, while a Th2 pattern is associated with progression to the severe disease form [7–11].

Current treatment for PCM relies on antifungal chemotherapy to control the disease. Clinically, the antifungal drugs most commonly used for PCM treatment include amphotericin B, sulfa derivatives, and azoles, but their toxicity can be a limiting factor in the treatment [12, 13]. Treatment regimens with these agents often require extended periods of maintenance therapy, which may range from months to years, and are usually associated with relapses [14].

There is a strong need for alternative clinical treatments to chemotherapy. Researchers have focused their efforts in investigating fungal components able to promote cellular immune responses and host protection. Immunization with heat-shock proteins (HSPs) from *P*. *brasiliensis* has been shown to provide some degree of protection against experimental disease [15–17]. The most abundant *P*. *brasiliensis* exocellular glycoprotein, called gp43, which is recognized by sera from virtually all *P*. *brasiliensis*- infected patients [18], was used to immunize mice. The whole gp43 molecule induces both CD4^+^ Th1 and Th2 cellular immune responses, whereas a 15-mer peptide derived from gp43, named P10, elicits IFN-γ-mediated Th1 immunity that protects mice from experimental PCM [19]. The therapeutic immunization of mice with a DNA vaccine encoding P10 and IL-12 inserts confers protection against experimental PCM, verified by reduced pulmonary fungal load [20].

It has been shown that *Saccharomyces cerevisiae* cell wall beta-glucan acts as an inherent adjuvant that activates dendritic cells required to elicit robust immune response [21]. In addition, previous studies showed that *S*. *cerevisiae* heat-killed yeast is able to protect mice against systemic coccidioidomycosis [22], by inducing both CD8^+^ and CD4^+^ Th1 responses in the infected mice [23]. Both responses were also activated when ovalbumin was carried as an antigen by *S*. *cerevisiae* [21].

On the basis of these observations, we hypothesized that *S*. *cerevisiae* could be a suitable vehicle for immunization against fungal infections. Here we evaluated the efficacy of immunizing mice against PCM by using *S*. *cerevisiae* yeast expressing gp43 as an immunogen.

## Material and Methods

### Animal Use and Ethics statement

The Committee for Ethics in Animal Research (CETEA) of the Ribeirão Preto Medical School, University of São Paulo (USP), approved all the procedures involving the use of mice, under the protocol 137/2008. BALB/c male mice, six- to eight-week-old, were housed under approved conditions in the institutional Animal Research Facilities. All animals were provided unlimited access to food and water. The animals were monitored daily for inspection of clinical signs. Euthanasia at the completion of experiments was carried out by carbon dioxide asphyxiation or cervical dislocation while under pentobarbital anesthesia.

### RNA extraction and production of the gp43 cDNA

Total RNA isolated from yeast cells of *P*. *brasiliensis* 18 was obtained by treatment with TRIzol reagent (Invitrogen Life Technologies, Carlsbad, CA, USA). The coding sequence of the gp43 antigen was obtained by RT-PCR. cDNAs were synthesized from total RNA from *P*. *brasiliensis* by using an oligo(dT) standard primer (0.5 μg) and a random primer reaction. The reaction was made using *Improm-II reverse transcriptase* (Promega Corporation, Madison, WI, USA), 5 μg of total RNA, buffer *Imrpom-II*-1x, 3 mM MgCl_2_, a mixture of dATP, dTTP, dCTP and dGTP (dNTPs) 0.5 mM to a final volume of 20 μL reaction.

### Construction of pBG1805_Gp43

The cDNA synthesized from the complete coding sequence of *gp43* was amplified using specific primers for cloning into the plasmid pCR 2.1-TOPO (Invitrogen Life Technologies, Carlsbad, CA, USA). The 5’ primers (5’ AGTACTATGAATTTTAGTTCTCTTAACCTGG 3’) had a direct initiator present in the sequence whereas the 3’ primers (5’ TCACCTGCATCCACCATACTT 3’) contained a stop codon. The PCR reaction was made to a final volume of 50 μL: Buffer Platinum *Taq* DNA polymerase *High Fidelity* (Invitrogen) (600 mM Tris-SO_4_ pH 8.9, 180 mM ammonium sulfate); 2 mM MgSO_4_; dNTPs 0.2 mM; 5 pmol of each primer and 1.25 units Platinum *Taq* DNA polymerase *High Fidelity*. The reaction included one cycle of 94°C (5 min), 29 cycles of 94°C (1 min), 50°C (1min), 68° C (1min) and one cycle of 68°C (10 min).

The PCR fragment generated a product with 1251 base pairs and was purified from the agarose gels by using the Kit GFX PCR DNA and Gel Band Purification (GE Healthcare). PCR products were cloned into plasmid pCR 2.1-TOPO (Invitrogen) and were sequenced in both directions.

The *gp43* ORF was amplified with other specific primers to recombine in the Gateway System (Invitrogen). The forward oligonucleotides contained 14 nucleotides derived from the attB1 site and 25 nucleotides contained in the *gp43* ORF near to the ATG translation initiator (5’-CAAAAAAGCAGGCTTCATGAATTTTAGTTCTCTTAACCTGG-3’). The reverse oligonucleotides contained 17 nucleotides from the *attB2* site and 18 nucleotides in the *gp43* ORF near the terminator site but did not carry the stop codon (5’ GTACAAGAAAGCTGGGTCCCTGCATCCACCATACTT- 3’). Oligonucleotides had additional nucleotides to maintain the open reading frame. The reaction included one cycle of 94°C (5 min), 24 cycles of 94°C (1 min), 51°C (1min), 68° C (1min) and one cycle of 68°C (10 min). PCR products were recombined into the Gateway vector pDONR 201 using BP Clonase (Invitrogen). The recombined products were transformed in *Escherichia coli*. After a plasmid extraction, ORF containing plasmid DNAs were recombined into a destination vector pBG1805 (described in (24)), using LR Clonase (Invitrogen). *E*. *coli* was transformed with the recombined plasmid pBG1805_Gp43. This plasmid was used to transform *Saccharomyces cerevisiae* (strain Y258). The resulting strain (yMAgp43) was used for protein overexpression.

### Expression and detection of the gp43 protein in *S*. *cerevisiae*

For overexpression in *S*. *cerevisiae*, the *gp43* ORF cloned into the vector pBG1805 was expressed as previously described [24]. Yeast cells were induced with YP +2% galactose medium (yeast extract 10 g/L, peptone 20 g/L, galactose 20 g/L). The ORF was expressed under control of the *GAL1* promoter with their C-terminus fused to a complex tag containing 6xHIS, HA epitope followed by a 3C site and the ZZ domain of protein A. After 4 and 16 hours of induction, the cells were recovered by centrifugation at 4000 rpm for 10 minutes at 4°C, washed and the pelleted cells suspended in PBS.

The induction of expression was monitored in 10% polyacrylamide gel by SDS-PAGE, and the preparation from total protein extract was done as previously described [24]. To confirm the expression, protein was electrotransferred from polyacrylamide gels to nitrocellulose membranes (Hybond-P, GE Healthcare Biosciences, Pittsburgh, PA, USA), at 75 V for two hours using transfer buffer (Tris-Base 1.895%, 9.09% Glycine). The membrane was rinsed with TBS-T 1X (Tris-HCl 60.5 g/L, NaCl 87.6 g/L, 0.05% Tween 20) with 5% skim milk, for 14–16 hours at 4°C, to block nonspecific interactions. Membranes were then incubated for 1 hour at room temperature, with anti-gp43 monoclonal antibodies (1:50), kindly provided by Ebert Hanna, or with monoclonal primary anti-HA (1:2000) (Santa Cruz Biotechnology, Santa Cruz, CA, USA) that reacts with the HA C-terminal tag. Three subsequent washes of 10 minutes were done with TBS-T plus 5% skimmed milk, at room temperature. The membranes were incubated with secondary antibody anti-mouse IgG conjugated with peroxidase (ECL western blotting reagent detection and Analysis System) diluted 1:4000 in TBS-T. After additional series of washes, development was done following manufacturer instructions (GE Healthcare).

Aliquots of 2x10^7^ recombinant yeasts expressing the gp43 protein (yMAgp43) were killed by heating at 56°C for 1 h and stored at -80°C until used for mouse immunization. The same procedure was done with yeasts carrying the empty plasmid (yMA).

### Vaccine administration

To evaluate the prophylactic effect of recombinant *S*. *cerevisiae* upon *P*. *brasiliensis* infection, groups of BALB/c mice were immunized intraperitoneally (i.p.) with 2x10^7^ yeast cells given weekly for three times (day 0, 7, and 14). These cells were previously harvested by centrifugation, suspended in PBS and killed by heating (56°C by 1h). One group was immunized with recombinant yeast expressing the gp43 protein (yMAgp43; Vaccine group), and the other two groups of mice were used as controls: one group was immunized with yeast cells carrying an empty plasmid (yMA; Vector Group) and the other with vehicle only (PBS Group).

### Cultivation of *P*. *brasiliensis*

Yeast cells from *P*. *brasiliensis* 18 strain were collected after growth in YPD liquid medium (Difco) at 37°C for 10 days. The viability of the yeasts were determined as previously described [25]. Pb18 strain was used based on its high virulence and ability to induce a strong granulomatous reaction [26].

### Experimental Infection

One week after the last immunization with yMAgp43, yMA or PBS, BALB/c mice were infected intravenously (i.v.) with 1x10^6^ *P*. *brasiliensis* yeast cells in 100 μL of PBS, through the ophthalmic plexus. The course of infection was evaluated 30 and/or 60 days post-infection.

### Histopathological examination of infected mice

Fragments of the right lobe of the lung from all mice, obtained on day 30 or 60 post infection, were fixed in 4% paraformaldehyde in 0.1 M phosphate buffer for 24 hours and processed for paraffin embedding. They were serially cut into 5-μm-thick-sections and sequentially stained with hematoxylin and eosin (H&E), for analysis of granulomatous lesions and inflammatory infiltrates, or with Grocott’s methenamine silver impregnation, to detect polysaccharides in the fungal cell wall based in an oxidation reaction. The granuloma count (number of granuloma/mm^2^) in lung sections was determined by using an optical microscope with an integrator lens (Carl Zeiss, Germany). The granuloma area (mm^2^) was measured by with a KS-100 program (Carl Zeiss, Germany).

### Assay for Organ Colony-Forming Units

Mice from all experimental groups were euthanized 30 or 60 days post-infection and fungal burden was measured by colony-forming units (CFU). Lung and splenic fragments were aseptically collected, weighed, homogenized in 1 mL of sterile PBS, and serially diluted. Aliquots of 100 μL were dispensed, in duplicates, into Petri dishes containing brain heart infusion agar (BHI, Difco Laboratories, Detroit, MI, USA), supplemented with 4% (v/v) heat-inactivated fetal bovine serum. After 14 days incubation at 37°C, the colonies were counted and the numbers of CFU per gram of tissue were calculated.

### Quantification of cytokines

To determine the organ contents of IL-12 and IFN-γ cytokines, lung and spleen homogenates were centrifuged at 2000 *x g* for 15 minutes, and the supernatants were analyzed by ELISA (OptEIA set; Pharmingen, San Diego, CA, USA), according to the manufacturer’s recommendations.

### Statistical analysis

Statistical differences between means of experimental groups were performed with analysis of variance (ANOVA) with GraphPad Prism5 version 3.01 using a post hoc Tukey test for multiple comparisons. Values were considered significant when p <0.05. All experiments were performed at least three times.

## Results

### Construction and Expression of recombinant gp43 in *S*. *cerevisiae*

**Fig 1:**
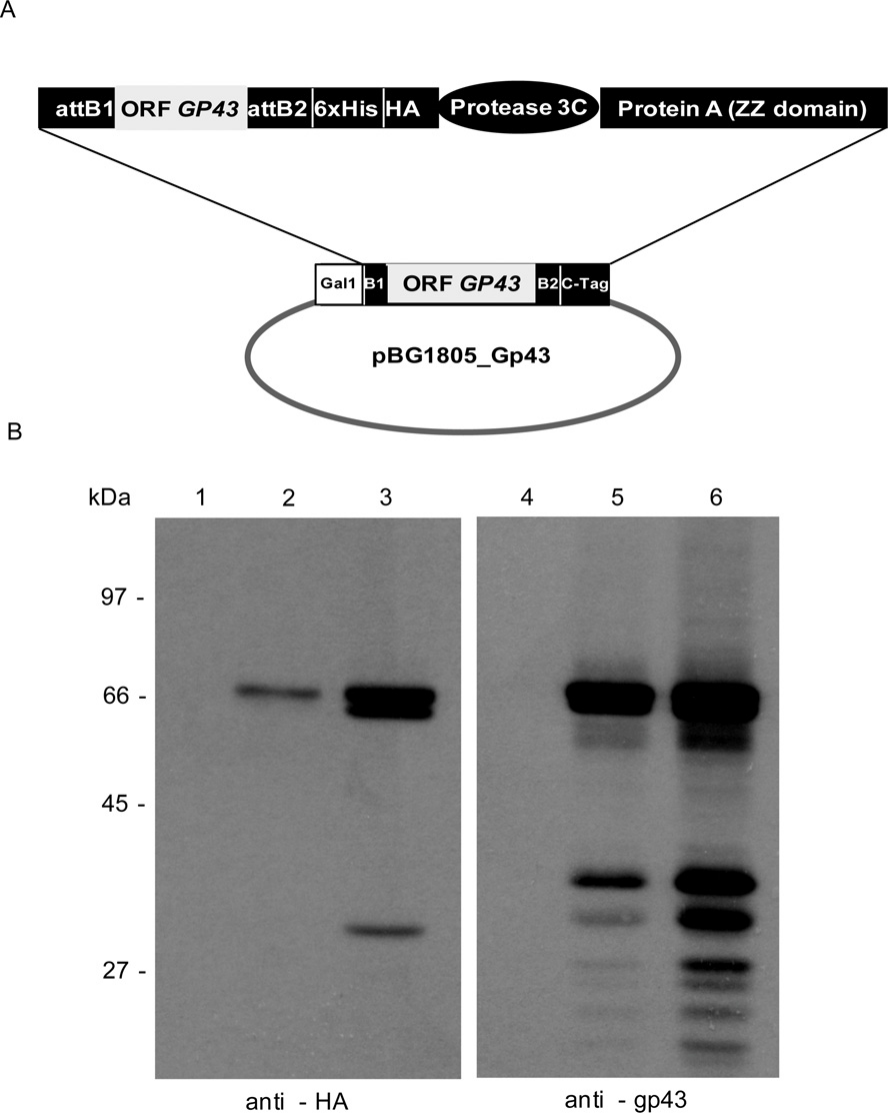
*Gp43* gene cloning and expression of the gp43 protein by the transformed *S*. *cerevisiae*. (A) Representative scheme of recombinant *gp43* cloned into the vector pBG1805 by Gateway system. The recombinant gp43 is fused to 6X His, HA, a cleavage site of protease 3C and ZZ domain of protein A in C-terminal site, besides being on promoter induction of *GAL1*. B1 and B2 are recombinant sites of the pBG1805. (B) Detection of recombinant gp43 protein by Western blotting. Overexpression of the gp43 fusion protein in *S*. *cerevisiae* yeast cells was induced by adding galactose to the YPD media. The cells grew for 4 hours (lanes 2 and 5) or overnight (3 and 6). Non-induced samples were used as controls (lanes 1 and 4). Total protein extracts were separated by SDS-PAGE, transferred to nitrocellulose membranes and reacted with anti-HA or anti-gp43 antibodies. A polypeptide of approximately 66 kDa, which is the predicted molecular weight of the gp43-tagged fusion protein, was detected either by an anti-HA (Fig. 1B, left) or anti-gp43 polyclonal antibodies (Fig. 1B, right). The bands of molecular weight lower than 66 kDa may correspond to degradation products.

To obtain the recombinant gp43 protein, the *gp43* ORF was amplified from TOPO_GP43. The amplified fragment with specific primers (GP43_F, GP43_R) was recombined into Gateway vector pDONR 201 using BP Clonase (Invitrogen). A homologous recombination reaction was taken with fragment encoding gp43 (pDONR 201_Gp43) and then cloned into the expression plasmid BG1805 using LR Clonase (Invitrogen). In the C-terminal portion of the recombinant plasmid pBG1805_Gp43, the protein gp43 was fused in tandem with 6X His, HA, a cleavage site of protease 3C and ZZ domain of protein A (Fig. 1A).

The recombinant plasmid pBG1805_GP43 was transformed into strain Y258 of *S*. *cerevisiae* Y258. Western blot analysis of the protein extracts of S. *cerevisiae* yeast cells expressing tagged gp43 (yMAgp43) at 4 and 16 hours after induction with galactose showed a band of apparent molecular mass of 66kDa, which corresponds to the predicted size of gp43 in fusion with a HA-His tag plus the protein A ZZ domain (Fig. 1B). This recombinant protein is detected either by anti-HA (Fig. 1B, left) or anti-gp43 polyclonal antibodies (Fig. 1B, right).

### Lung Histopathology

**Fig 2:**
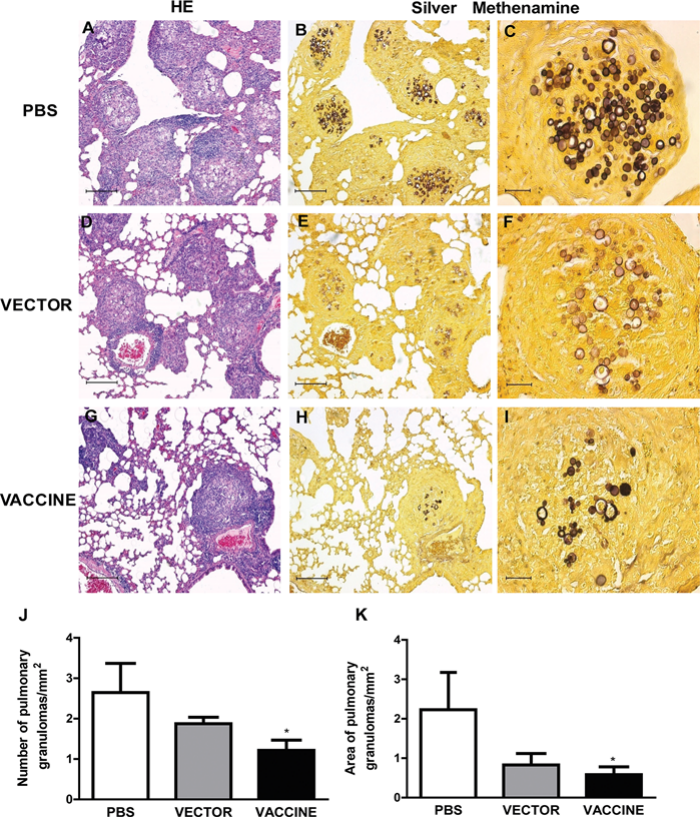
Pulmonary histopathology and morphometric analysis of the granulomas. The pulmonary histopathology of immunized mice infected with *P*. *brasiliensis* were analyzed thirty day post infection. Lungs of mice immunized with yMAgp43 (Vaccine Group) presented discrete granulomatous infiltrates (HE, 10X, G) if compared to controls (Vector and PBS Groups, D and A). Silver methenamine staining revealed that the lungs in the Vaccine Group (H and I) presented less yeast cells and granulomas when compared to controls (B, C, E and F). Panel J: The number of granulomas/mm^2^ in the tissue was obtained using an optical microscopy with the aid of an integrator lens (Carl Zeiss Jena, Germany). Each bar represents the average ± SD of lung sections from 4 animals. Panel K: the granuloma area (mm^2^) was determined by means of KS-100 program (Carl Zeiss Jenamed 2, USA). The data represent the average ± SD of lung sections from 4 animals, where: *P < 0.05 in relation to the PBS group.

To evaluate whether prophylactic administration of *S*. *cerevisiae* expressing gp43 affects the course of experimental PCM, groups of BALB/c mice were inoculated i.p. with recombinant yeast expressing the gp43 protein; with yeast cells carrying an empty plasmid; or with vehicle only, on days 0, 7, and 14. Challenge was performed one week after the third immunization, by i.v. inoculation, with *P*. *brasiliensis* yeast. Thirty days after infection, the lungs were examined for inflammation and fungal presence, by staining the tissue\ sections with hematoxylin and eosin (HE), or with silver methenamine. The lung parenchyma of PBS group mice was largely replaced by large and loose granulomas (Fig. 2A). In the rare preserved areas, thickening of alveolar septa were observed. The granuloma, in its central area, presented phagocytic cells ordered around numerous yeasts (Fig. 2B and 2C). The presence of yeast cells were extended to the periphery of some granulomas, an area that is characterized by intense basophilia due to the presence of plasmocyte nuclei. Some budding yeasts were seen inside the granulomas (Fig. 2B and 2C). The lung of mice of the empty vector group exhibited less inflammatory infiltrates and fewer granulomas (Fig. 2D), when compared to the PBS group (Fig. 2A), differences that were not confirmed by the morphometric analysis (Fig. 2J and 2K). Notably, in the Vaccine group, lung architecture was more extensively preserved, showing thin alveolar septa. In the fewer and more compact granulomas, yeast cells were less frequent and restricted to the central area of the granuloma (Fig. 2G-I).

The pulmonary morphometric analysis showed that vaccinated mice, compared to the controls, had a lower number of granulomas (Fig. 2J), which occupied a smaller parenchymal area (Fig. 2K). On the other hand, there was no significant difference between empty vector group and PBS group regarding the density of granulomas and the area they occupied. Notably, the PBS group presented 2.64 granulomas/mm^2^, whereas the vaccine group presented 1.21 granulomas/mm^2^ (Fig. 2J), corresponding to a 54% reduction in the granuloma number. Besides the lower number of granulomas found in mice of the vaccine group, the total area occupied by the granulomas was significantly lower than that verified in the PBS group (Fig. 2K). The granulomas of the vaccinated mice occupied the total area of 0.58mm^2^, which is 3-fold lower than the total area occupied by granulomas in control animals. These results show that the vaccination with *S*. *cerevisiae* expressing the gp43 recombinant protein promoted a significant reduction in the pulmonary damage caused by *P*. *brasiliensis* infection.

### Fungal burden in immunized mice

**Fig 3:**
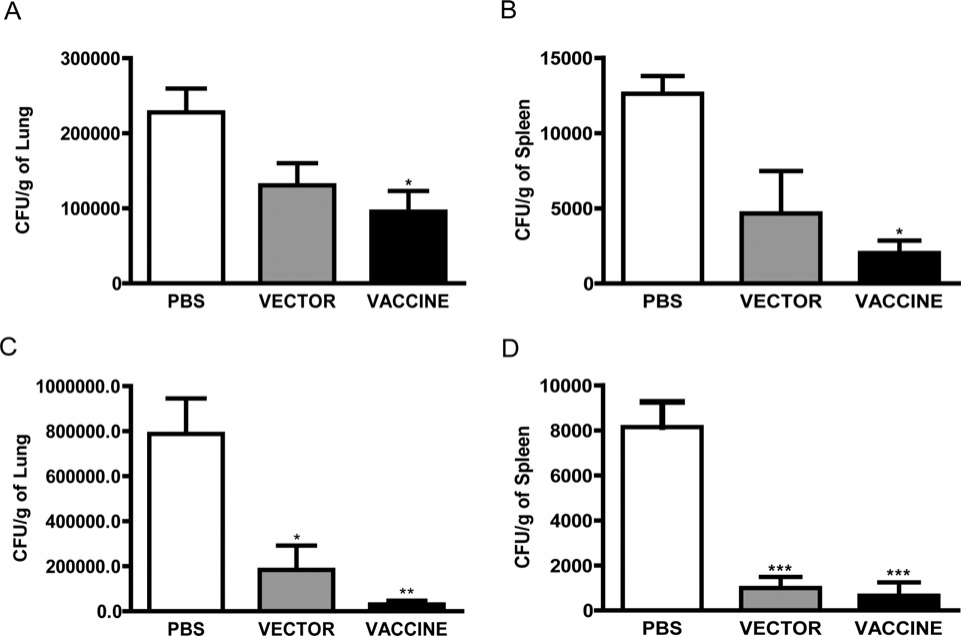
Quantification of fungal burden in the lung and spleen of recombinant yeast-immunized mice, after infection with 1x10^6^ yeasts of *P*. *brasiliensis*. Bars represent CFU levels in lungs (A and C) and spleens (B and D). BALB/c mice were intraperitoneally inoculated with PBS (PBS group), or yMA (Vector Group), or yMAgp43 (Vaccine group) and intravenously infected with *P*. *brasiliensis*. One and two months after infection, all mice were sacrificed. The Vaccine group mice presented reduced levels of colony forming units (CFU) when compared with the PBS groups in both organs and both time (A-D). In addition, sixty days post infection, the CFU number in the Vector Group mice was significantly lower than PBS group (C and D). Data are representative of a typical experiment independently repeated three times, where: * P<0.05, **P<0.01, and ***P<0.001 in relation to the PBS Group.

In order to determine whether vaccination confers protection against *P*. *brasiliensis* infection, lung and spleen fragments of immunized and control (Vector and PBS groups) mice, challenged with 1X10^6^ *P*. *brasiliensis* yeasts, were obtained 30 or 60 days post-infection and analyzed for fungal CFU. On day 30 post-infection, the number of CFU recovered from the organs of mice from the vaccine group was significantly lower than that of the PBS group. The reductions attained 50% and 84% in the lung and in the spleen, respectively (Fig. 3A and 3B). These differences were more pronounced on day 60 post-infection, and extended to the empty vector group, whose fungal burden was 4- and 8-fold lower than the provided by the lung and the spleen of mice from the PBS group. As on day 30 post infection (Fig. 3A and 3B), the protection conferred by the yeast expressing gp43 was higher than that achieved by yeast with the empty vector group on day 60 post-infection (Fig. 3C and 3D). The observed reductions in fungal load indicate that vaccine promoted efficient *P*. *brasiliensis* yeasts clearance, which is importantly favored by the adjuvant effect provided by *S*. *cerevisiae*.

### Cytokines content in organs of immunized mice

**Fig 4:**
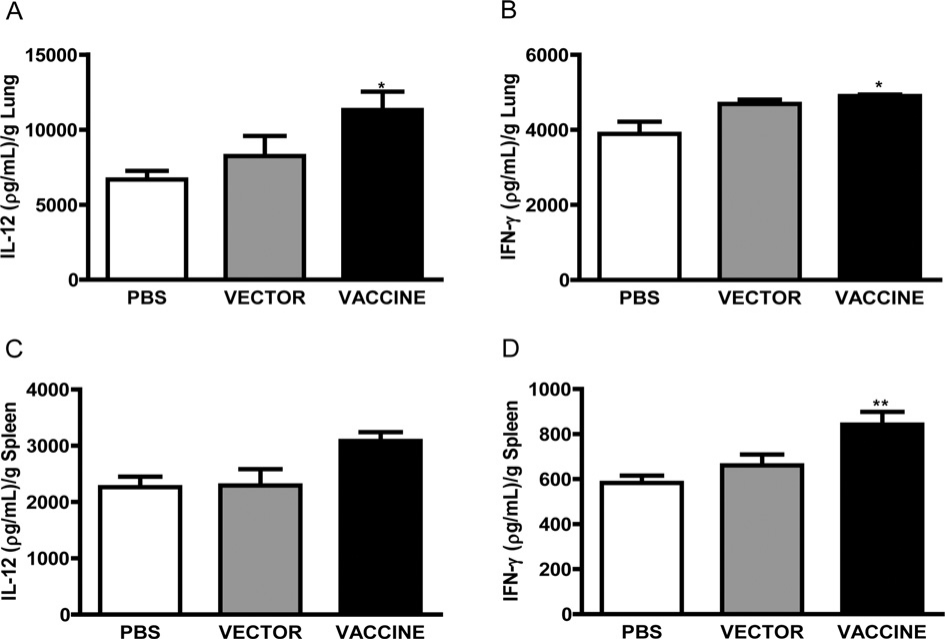
Quantification of cytokines in the lung (A and B) and spleen (C and D) of mice inoculated (i.p) with PBS, or yMA, or yMAgp43, and infected (i.v.) with 1x10^6^ yeasts *P*. *brasiliensis*. Bars represent levels of IL-12p40 (A and C) and IFN-γ (B and D) detected in the organs of mice from different experimental groups at 30 days post-infection. All immunized mice (Vaccine group) showed higher levels of IL-12 and IFN-γ, in both organs, when compared to controls (PBS). Data are representative of a typical experiment independently repeated three times, where: * P<0.05, and **P<0.01 in relation to the PBS Group.

To investigate the mechanism by which immunization with yMAgp43 has conferred protection against *P*. *brasiliensis* infection, the levels of cytokines contained in the organs of vaccinated (yMAgp43) and control mice (Vector and PBS) were quantified 30 days after fungal challenge. Significantly higher concentrations of IL-12 were detected in the pulmonary tissue, and of both IL-12 and IFN-γ, in the pulmonary and splenic tissues of mice vaccinated with yMAgp43 in comparison to those found in mice of either control group (PBS or Vector) (Fig. 4). This finding suggests that the vaccination of mice with yMAgp43 elicited a Th1-immune response, which is known to account for protection against the fungus. The slightly higher cytokine content observed in the vector group compared to PBS may be due to the adjuvant effect conferred by yeast. This is consistent with protection presented by the vector group as evaluated by histopathology and CFU measurements (Figs. 2 and 3). The significantly higher protection conferred by the vaccine group (yMAgp43) may be a result of a specific response against gp43 in combination with the yeast adjuvant effect.

## Discussion

In this study we show that immunization with *S*. *cerevisiae* expressing the gp43 recombinant protein (yMAgp43) prior to challenge with *P*. *brasiliensis* protects mice against systemic PCM. This protection was revealed by more effective fungal clearance from the lungs of immunized mice, as well as by reduction in density of pulmonary granulomatous lesions, associated with the presence of few viable yeast. These beneficial effects are likely due to the development of Th1 immunity, featured by augmented pulmonary levels of IL-12 and IFN-γ.

The analysis of the pulmonary tissue of *P*. *brasiliensis* infected mice showed that those mice vaccinated with yMAgp43 had less frequent and more compact granulomas, which are the prominent inflammatory lesions in human and experimental PCM, and known to restrict fungal growth and avoid dissemination throughout the host organism [27, 28]. These roles are not played by loose granulomas, which were extensively found in non-vaccinated mice. In addition, vaccinated mice showed fewer silver stained fungi in the center of pulmonary granulomas, a finding that is consistent with the significantly lower fungal burden presented by these mice in comparison those of the control groups, and reflects the protection level provided by the vaccination procedure. Moreover, the protection conferred by the immunization with yMAgp43 was equivalent to the previously reported for immunization with gp43 or its derived 15 amino-acid peptide, named P10, when administered in association with adjuvants [19]. Administration of the non-expressing gp43 *S*. *cerevisiae* yeast (Vector group) did not reduce significantly the granulomas density nor the fungal burden, examined at the 30^th^ day post-infection. However, at the 60^th^ day the empty vector group showed fungal burden as low as the vaccinated group, a result that is consistent with the reported use of non-recombinant *S*. *cerevisiae* yeasts as a vaccine against coccidioidomycosis and other fungal infections [22]. As a result of its adjuvant properties, *S*. *cerevisiae* yeast may induce a certain grade of protection, as provided by therapy with complete Freund's adjuvant against murine PCM, and the protection may be induced by cross-protective antigens [29].

Extensive studies have shown that *S*. *cerevisiae* successfully delivers proteins into dendritic cells [21, 30–32], which are uniquely able to shape antifungal immunity by initiating naïve T cell responses [33, 34]. The stimulus of IL-12 produced by activated dendritic cells accounts for the development of Th1 effector cells [35], which release high concentration of IFN-γ This cytokine, in turn, induces the antifungal effector function of phagocytes. As a result, this sequence of events explains why a dominant Th1 response correlates with the occurrence of protective immunity to fungal infection [34]. Because we found significant increase of the IL-12 and IFN-γ contents in organs of the vaccinated mice when challenged with *P*. *brasiliensis*, we postulate that mice immunized with *S*. *cerevisiae* yeasts expressing the gp43 antigen have used efficiently the mechanisms mentioned before, involved in resistance against PCM.

Our postulation is supported by studies showing that severe PCM in humans and experimental hosts is associated with depression of cell-mediated immune responses [2, 36], while resistance to *P*. *brasiliensis* is often related with the production of IFN-γ [9, 37–39]. Indeed, IFN-γ production is required for resistance against several infections by pathogenic fungi, such as *Cryptococcus neoformans* [40, 41], *Histoplasma capsulatum* [42], *Blastomyces dermatitidis* [43]. Furthermore, the protective effect against *P*. *brasiliensis* infection conferred by immunization with the peptide P10 was related to the *in vitro* demonstrated peptide ability of inducing an IFN-γ secreting Th1-lymphocyte population [19].

Immunotherapy with P10 has been effective using adjuvants, P10-primed dendritic cells, and especially a combination of plasmids encoding P10 and IL-12 gene [44], in a demonstration that an additional stimulus to induce T cells, beyond the peptide itself, is necessary to confer protection against the fungus. We hypothesize that in our study the protection induced by immunization with the whole gp43, which encompasses P10 and several Th2 epitopes as mapped by Taborda et al. (1998), was made possible by the ability of the *S*. *cerevisiae* as a vehicle to support Th1 immunity.

Heat-killed *S*. *cerevisiae* has been successfully used as a vaccine against several fungal infections, such as systemic aspergillosis, coccidioidomycosis, candidiasis and cryptococcosis [22, 45–47]. The immune response elicited by the heat-killed yeast is characterized mainly by high IFN-γ production, which is similar to that obtained by administration of live yeasts [23] and coincident with the already mentioned profile detected here in mice immunized with the *S*. *cerevisiae* expressing gp43. Since the *S*. *cerevisiae* yeast itself also conferred a significant protection against *P*. *brasiliensis* infection, it is reasonable to envisage further studies to better explore the eventual resistance that may be conferred by *S*. *cerevisiae* derived preparations.

In summary, we tested a new approach to immunoprophylaxis against PCM that uses *S*. *cerevisiae* as a vaccine vehicle. Further yeast vaccine refinements may be implemented for use as a therapeutic tool against PCM.
